# A 40 Mb/s VLC System Reusing an Existing Large LED Panel in an Indoor Office Environment †

**DOI:** 10.3390/s21051697

**Published:** 2021-03-02

**Authors:** Xicong Li, Zabih Ghassemlooy, Stanislav Zvánovec, Paul Anthony Haigh

**Affiliations:** 1 Optical Communications Research Group, Faculty of Engineering and Environment, Northumbria University, Newcastle upon Tyne NE1 8ST, UK; z.ghassemlooy@northumbria.ac.uk; 2 Department of Electromagnetic Field, Faculty of Electrical Engineering, Czech Technical University in Prague, 16627 Prague, Czech Republic; xzvanove@fel.cvut.cz; 3 Intelligent Sensing and Communications Group, School of Engineering, Newcastle University, Newcastle upon Tyne NE1 7RU, UK; paul.haigh@newcastle.ac.uk

**Keywords:** visible light communication, LED, carrierless amplitude and phase modulation (CAP), LED driver, bias-tee, lighting infrastructure

## Abstract

With advances in solid-state lighting, visible light communication (VLC) has emerged as a promising technology to enhance existing light-emitting diode (LED)-based lighting infrastructure by adding data communication capabilities to the illumination functionality. The last decade has witnessed the evolution of the VLC concept through global standardisation and product launches. Deploying VLC systems typically requires replacing existing light sources with new luminaires that are equipped with data communication functionality. To save the investment, it is clearly desirable to make the most of the existing illumination systems. This paper investigates the feasibility of adding data communication functionality to the existing lighting infrastructure. We do this by designing an experimental system in an indoor environment based on an off-the-shelf LED panel typically used in office environments, with the dimensions of 60 × 60 cm${}^{2}$. With minor modifications, the VLC function is implemented, and all of the modules of the LED panel are fully reused. A data rate of 40 Mb/s is supported at a distance of up to 2 m while using the multi-band carrierless amplitude and phase (CAP) modulation. Two main limiting factors for achieving higher data rates are observed. The first factor is the limited bandwidth of the LED string inside the panel. The second is the flicker due to the residual ripple of the bias current that is generated by the panel’s driver. Flicker is introduced by the low-cost driver, which provides bias currents that fluctuate in the low frequency range (less than several kilohertz). This significantly reduces the transmitter’s modulation depth. Concurrently, the driver can also introduce an effect that is similar to baseline wander at the receiver if the flicker is not completely filtered out. We also proposed a solution based on digital signal processing (DSP) to mitigate the flicker issue at the receiver side and its effectiveness has been confirmed.

## 1. Introduction

Visible light communication (VLC) has attracted continued research interest [1,2,3], while solid-state lighting has dominated the mass market over the last two decades [4]. The use of solid-state semiconductor devices, such as light-emitting diodes (LEDs), organic light-emitting diodes (OLEDs) [5,6,7], and laser diodes (LDs) [8,9], in the visible range enables intensity modulation of the optical power for communication simultaneously with illumination. Therefore, the VLC technology has the potential to reuse the existing lighting infrastructure to deliver add-on functionality in a cost-effective manner that can be adopted in future smart indoor and outdoor environments. The outdoor lighting infrastructure covers a variety of LED-based luminaries that range from street lights, traffic lights, to car lights, which have inspired the use of VLC in infrastructure-to-vehicular (I2V) and vehicular-to-vehicular (V2V) communications [10,11]. Experiments using existing traffic lights and car lights as transmitters have been reported to demonstrate the feasibility of VLC in the outdoor I2V/V2V communications [12,13]. When compared with the outdoor environment, the indoor scenarios often find large LED luminaries installed in the ceiling or relatively small LED desk lamps placed directly above the table to provide a guaranteed light level for illuminance. This benefits the indoor VLC applications greatly since those LED luminaries’ high output optical power can ensure a decent signal-to-noise (SNR) profile within rooms/offices if their intensity is efficiently modulated. In this work, we hereafter limit the scope to VLC deployment in the indoor environment because of its advantages and popularity.

Being motivated by the potential of VLC, standardisation has been carried out by several organisations [14,15,16]. Recent standardisation activities are mainly conducted within the Institute of Electrical and Electronics Engineers (IEEE) and the International Telecommunication Union Telecommunication Standardisation Sector (ITU-T) [16]. In March 2017, the IEEE 802.15.13 task group was established with the goal of defining an optical wireless communication (OWC) standard that supports data rates of up to 10 Gb/s using wavelength that ranged from 190 to 10,000 nm. The first draft was finished in 2019 with a letter ballot held in 2020. The standard is expected to be completed in 2021. In parallel with 802.15.13, the IEEE 802.11bb task group was formed in May 2017 to develop a global OWC standard targeted at the mass market within the well-known IEEE 802.11 wireless local area networks (WLAN, which is often linked and used interchangeably with WiFi, i.e., wireless fidelity) standard family. Because the latest standards typically define communications operating in the band beyond the visible light spectrum, the concept of light fidelity (LiFi), which covers both visible and infrared bands, has been introduced [17]. The ambition of 802.11bb is to operate as closely as possible to the base 802.11 standards to allow for integration with WLAN chipset vendors. Consequently, the physical (PHY) layer of 802.11a with a bandwidth of 20 MHz has been adopted as the common PHY mode in 802.11bb. The draft is still under discussion and expected to be completed in 2021. As another influential standardisation body, ITU-T published a new VLC/LiFi standard G.9991 (also known as G.vlc) [18] in 2019, which is highly based on the ITU-T’s high-speed power-line communication standard G.9960 (also known as G.hn) [19,20]. In the PHY layer, G.9991 defines two PHY modes that employ the orthogonal frequency division multiplex (OFDM) modulation with three band plans. The total bandwidth in the three band plans is 50, 100, and 200 MHz, respectively. A maximum data rate of 2 Gb/s is supported. Since the release of G.9991, several chipset implementations have been available from the G.hn semiconductor vendors, helping to accelerate VLC/LiFi applications in the real world [16].

Nevertheless, not much attention has been paid to the viability and complexity of implementing VLC using the existing lighting infrastructure following the standardised specifications. The current commercial products normally require the complete replacement of the existing illumination systems with new luminaires supporting VLC. It is therefore desirable to make the most of the existing illumination systems to save the investment and reduce the deployment cost. In this paper, we investigate the possibilities and challenges in upgrading the existing LED-based luminaries with VLC functionality by developing a demonstration system in a practical indoor environment. To the best of the authors’ knowledge, there exist limited reports on reusing the existing commercial LED luminaries for simultaneous VLC communication and illumination in the literature. In [21], a commercial LED table lamp was upgraded to a VLC system providing a 10Base-T Ethernet connection via an optical wireless transmission of 12.5 Mb/s Manchester coded on-off-keying (OOK) signals. The achievable system capacity is not fully exploited due to the transmission speed limitation of the Ethernet connection. Additionally, challenges and issues in implementing such a practical system based on existing luminaries are not covered in [21].

Most of the indoor VLC systems that are reported in the literature are based on dedicated designs using LEDs over a short distance in a controlled laboratory environment, focusing on achieving high data rates. Recently reported experimental systems have been demonstrating transmission speed in the order of Gb/s with precisely aligned point-to-point links that are based on single-colour LEDs (scLEDs) [22,23,24]. The use of the scLEDs significantly contributes to the throughput in Gb/s, since they offer a higher modulation bandwidth than the phosphor-converted white LEDs (pcwLEDs) with the absence of the slow response phosphor. However, the existing illuminance products have been widely adopted pcwLEDs due to their low cost, thus imposing hurdles to achieving comparable date rates. As shown later, the LED panel that was adopted in our demonstration system consists of 240 pcwLEDs and it has a 3-dB bandwidth of only 1.4 MHz. Although the bandwidth of pcwLEDs can be increased to between 10 and 20 MHz by using blue filters at the receiver side to mitigate the slow temporal response of the phosphor, this improvement in bandwidth is paid for at the cost of power loss or signal-to-noise ratio (SNR) penalty due to the phosphor-converted portion of the received light spectrum being filtered out [25]. It is unnecessary to use blue filters if multi-carrier modulation schemes, especially with bit-loading, are applied because multi-carrier modulation can utilise all of the signal power in the full received light spectrum to achieve higher SNR and, therefore, higher system capacity, or data rates [26]. Similarly, analogue pre-equalisers [27,28] are designed to compensate for the frequency response of the LED to extend the normalised 3-dB bandwidth by sacrificing the modulation depth, which leads to the same issue as using blue filters [29]. Hence, we adopted a multi-band carrierless amplitude and phase modulation (CAP) [30,31,32] with bit-loading to combat the limited bandwidth problem due to its simple implementation and low peak-to-average power ratio (PAPR) [33].

In addition, those high-speed experimental VLC systems often require bulky optics to realise precise alignment between transmitters and optical receivers, which is unreasonable for practical VLC applications in the real world. An optical receiver with a large field of view (FoV) and sensitivity is a key enabling factor for the deployment of VLC applications. We have designed and built a compact optical receiver with a half angle of ±35${}^{\circ }$, which highly relaxes the alignment requirement, in order to demonstrate the usability of our system in real-world scenarios.

Another key challenge that is faced in our system is the flicker caused by the low-cost LED driver. The residual current ripple from the LED driver introduces unwanted low-frequency light intensity modulation and, hence, reduces the modulation depth of VLC signals. Optimised LED driver circuits for VLC have been proposed to address the flicker issue [34,35]. In this paper, we still use the existing LED driver with the flicker problem in order to fully reuse the existing illumination system and keep the modification within a minimum degree. The detrimental effect of flicker on a binary phase modulation (BPM) VLC system is investigated in [36] under the condition that the flicker is treated as a random interference. In this paper, we treat the flicker as an underlying stationary and predictable signal and, therefore, we can use a low-pass finite impulse response (FIR) filter to filter out the low-frequency components as an estimate of the flicker. After subtracting the estimated flicker from the received raw signal, the adverse effects of flicker can be greatly alleviated, offering a different approach to addressing the flicker at the receiver. Because the estimation of the flicker can be realised by a programmable FIR filter in the digital domain, this digital signal processing (DSP)-based solution can be universally applied to deal with a variety of flicker specifications.

Our demonstration system can support a data rate of 40 Mb/s at a distance of up to 2 m with a minimum illuminance level of 300 lux [37]. The VLC functionality is added to the existing light infrastructure without any degradation in the lighting level, presenting a usable VLC/LiFi system in an indoor office environment.

The remainder of this paper is organised, as follows. In Section 2, the demonstration system is described in detail. Section 3 discusses the key challenges in the implementation of the proposed system and presents the experimental results. Finally, conclusions are made in Section 4.

## 2. Experimental Setup

**Figure 1:**
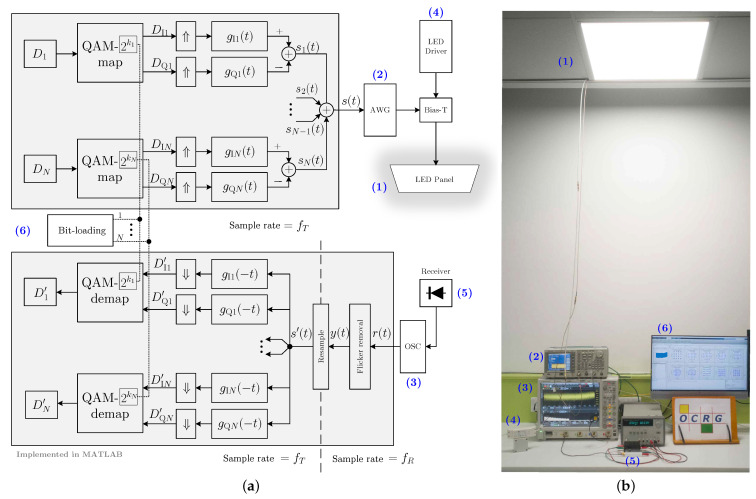
(**a**) System block diagram; (**b**) experimental setup.

Figure 1 shows the proposed system block diagram and a photo of the experimental setup. The lighting device used in our demonstration system is a 60×60 cm${}^{2}$ commercial LED panel (DFx 563-004-01), which has been installed in our laboratory ceiling. We use a bias-tee circuit to modulate the LED panel’s intensity with information-carrying signals being generated by an arbitrary waveform generator (AWG) and place an optical receiver underneath that is connected to an oscilloscope (OSC) to capture the regenerated transmitted signals for offline MATLAB processing. Full characterisation of the panel was performed, followed by modulation circuit design, modulation scheme design, and experimental test, to upgrade the LED panel with the data transmission function.

### 2.1. The Light-Emitting Diode (LED) Panel

**Figure 2:**
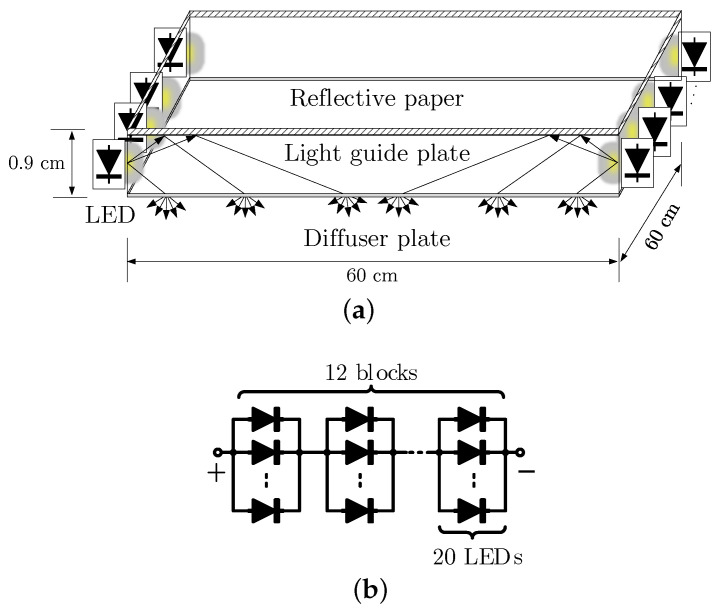
(**a**) Illustrative view, and (**b**) schematic of the light-emitting diode (LED) panel in use.

In Figure 2a, the internal structure of the LED panel is depicted. Two LED strips are mounted on opposite sides of a square aluminium frame that hosts a transparent light guide plate to direct the light evenly within the panel area. On the backside of the light guide plate is a single layer of reflective paper that is used to redirect the optical power to the receiver plane. The other side of the light guide is attached to a diffuser plate to produce evenly-distributed light for illumination. The two LED strips consist of 12 serially connected blocks of 20 LEDs that are connected in parallel, see Figure 2b.

### 2.2. LED Panel Driver and Characteristics

**Figure 3:**
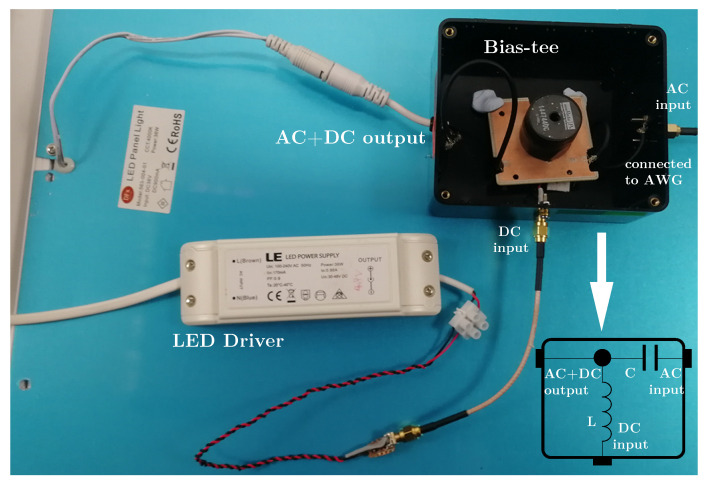
The bias-tee combining the LED driver and arbitrary waveform generator (AWG) to drive the LED panel.

**Figure 4:**
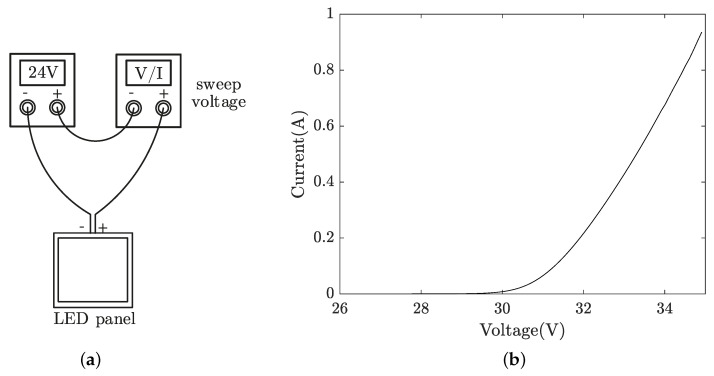
(**a**) Setup for *V*-*I* measurement; (**b**) measured *V*-*I* curve of the LED panel.

We utilised the existing LED panel driver and designed a bias-tee circuit, as illustrated in Figure 3, where the AC port of the bias-tee module is connected to an AWG for intensity modulation of the LED panel. Alternative LED drivers with low ripple/noise can be used, depending on the system requirements, but with increased cost and complexity. To measure the *V*-*I* curve of the LED panel, two low-noise voltage sources were connected in series, as shown in Figure 4a, to generate high enough voltages to turn on the LED strips. Because the turn-on voltage of each LED is around 2 V, the turn-on voltage for the entire LED string can be estimated to be ∼24 V. Therefore, the first voltage source is set to a fixed voltage of 24 V, while the second is set to sweep voltage and monitor the corresponding current. The measured *V*-*I* curve of the LED panel as a lumped element is shown in Figure 4b. From the datasheet, the LED panel is biased at 0.9 A, with a corresponding voltage of around 34.5 V.

**Figure 5:**
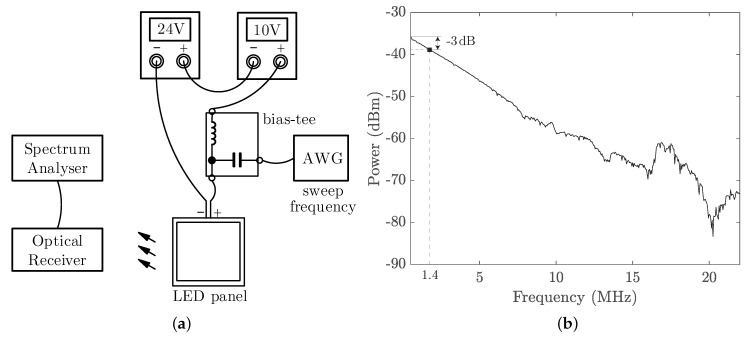
(**a**) Setup for frequency response measurement; (**b**) measured frequency response of the LED panel.

Next, we measured the frequency response of the LED panel at the bias point of (34.5 V, 0.9 A) using the setup that is shown in Figure 5a. Here, we used two low-noise voltage sources connected in series as in the *V*-*I* measurement to mitigate the light intensity flicker that is caused by the residual current ripple of the original LED panel driver. With an AWG and a spectrum analyser (SA), the frequency response is measured and plotted in Figure 5b with a 3-dB bandwidth of 1.4 MHz. Table 1 provides the list of equipment for characterising the LED panel.

### 2.3. Optical Receiver

**Figure 6:**
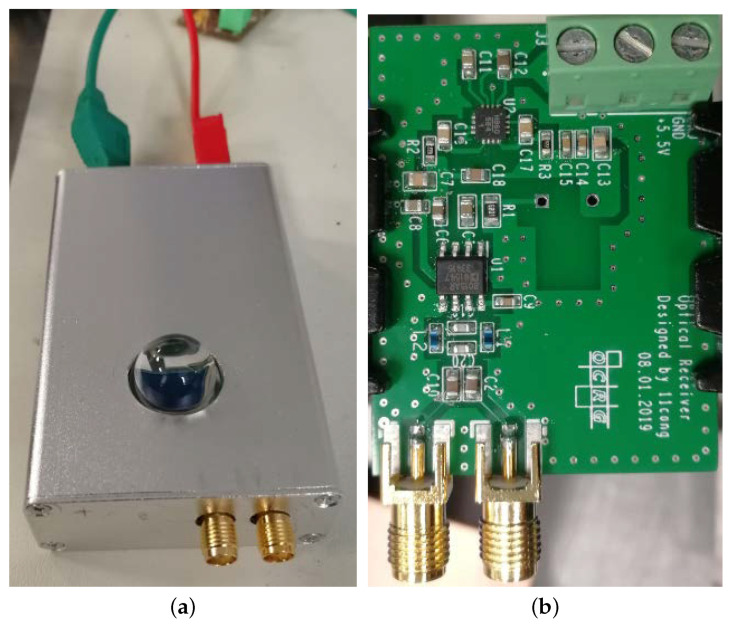
(**a**) Optical receiver and (**b**) the printed circuit board (PCB) inside.

We adopted a silicon PIN photodiode (Hamamatsu S6968) with a large effective photosensitive area of 150 mm${}^{2}$ and a built-in 14 mm (diameter) lens in the plastic package to relax the alignment requirement between the LED panel and the receiver. A trans-impedance amplifier (TIA) circuit converts the detected current from the photodiode into a voltage signal with a gain of 10 kΩ for the following data acquisition by the OSC. The optical receiver was implemented compactly using a printed circuit board (PCB) inside an aluminium enclosure. A photo of the optical receiver is given in Figure 6 with a closeup of the PCB inside.

**Figure 7:**
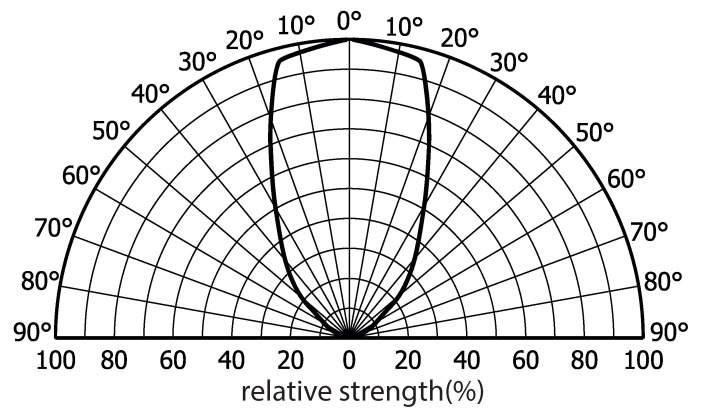
Measured directivity of the optical receiver.

Figure 7 depicts the receiver’s relative signal strength at varying incident light angles, from which a half angle of ±35${}^{\circ }$ can be observed. Here, the half angle is defined as the incident angle at which the strength is reduced to half of that generated when the incident light is perpendicular to the photodiode. We also observed that the optical receiver works robustly as long as the LED panel shines it in our practical measurements. In addition, using a high-bandwidth laser diode (Osram, PL450B) as the light source, we measured the 3-dB bandwidth of our receiver as around 20 MHz, which satisfies our system specification.

### 2.4. MultiCAP for the Downlink Visible Light Communication

It is well known that LEDs, especially those that are used for illumination, suffer from low 3-dB bandwidth (up to a few MHz) and decaying frequency responses [38], as shown in Figure 5b. Unlike radio frequency (RF), wireless technologies where multi-path induced fading is highly critical, multi-path fading is not a major concern and it poses limited impact on the overall system frequency response in indoor VLC systems. One main reason is that most practical indoor VLC systems are static line-of-sight (LOS) channels. The other reason is that the coherence bandwidth of a tpicyal indoor non-LOS (NLOS) channel is much higher than the LED’s 3-dB bandwidth, thus making the LED capable of approximately representing the frequency response of the entire VLC system. Recent experimental results have shown that a maximum root mean-square (RMS) delay spread of 14.2 ns was measured for a 3 m NLOS link in a worst practical indoor scenario [39,40]. The coherence bandwidth can be estimated to be in the order of several tens of MHz, which is still much higher than the LED panel’s bandwidth of 1.4 MHz that was used in this work. A multi-carrier version of carrierless amplitude and phase (MultiCAP) modulation with bit-loading is adopted to overcome the unideal channel response [41]. When compared with OFDM, MultiCAP has a lower peak-to-average power ratio (PAPR), because typical MultiCAP implementations tend to use a smaller number of carriers. In addition, the modulation and demodulation of MultiCAP can be implemented with reduced complexity, due to the absence of fast Fourier transform (FFT) and inverse FFT operations. The parameters and algorithms for the modulation and demodulation are described, as follows.

Suppose that a full band of $B_{\mathrm{mod}}$ is divided evenly into *N* sub-bands. Each sub-band is transmitting a band-pass CAP signal, which is similar to quadrature amplitude modulation (QAM). For example, the nth sub-band is centred at the carrier frequency $f_{n}=\frac{2n-1}{2N}B_{\mathrm{mod}}$, where n=1,⋯,N. Because the band is divided evenly, the baseband symbol rate $R_{s}$ in each sub-band is the same and is equal to $\frac{B_{\mathrm{mod}}}{N(1+\beta )}$ when using the square-root raised cosine (SRRC) filter with a roll-off factor of β for pulse shaping. The MultiCAP filter bank consists of *N* pairs of pulse shaping filters, as given by:

(1) $$\begin{array}{cc} g_{In}\left(t\right) & =g\left(t\right)\cos2\pi f_{n}t \end{array}$$

(2) $$\begin{array}{cc} g_{Qn}\left(t\right) & =g\left(t\right)\sin2\pi f_{n}t \end{array}$$

where

(3) $$g\left(t\right)=\frac{\sin\left[\pi \left(1-\beta \right)t/T_{s}\right]+4\beta t/T_{s}\cos\left[\pi \left(1+\beta \right)t/T_{s}\right]}{\pi t/T_{s}\left[1-{\left(4\beta t/T_{s}\right)}^{2}\right]}$$

is the SRRC pulse and $T_{s}=1/R_{s}$ is the baseband symbol period. The synthesis of pulse shaping filters in (1) with built-in carriers is the major difference between CAP and QAM. Because of this feature, CAP does not require extra free-running oscillators and mixers to shift the spectrum to the target frequency range, which is beneficial when designing low-cost systems.

Block 6 of Figure 1a illustrates the schematic diagram for the implementation of modulation and demodulation in MultiCAP. At the transmitter, for the nth sub-band, a random symbol sequence $D_{n}$ is split into two branches, i.e., an in-phase (I) sequence $D_{In}$ and a quadrature (Q) sequence $D_{Qn}$, after the QAM-$2^{k_{n}}$ constellation mapping. Here, $k_{n}$ denotes the QAM constellation order (in bits) that is used in the nth sub-band. Subsequently, the baseband symbol sequences $D_{In}$ and $D_{Qn}$ are up-sampled and filtered by the nth in-phase and quadrature filters of $g_{In}\left(t\right)$ and $g_{Qn}\left(t\right)$, respectively. Finally, the nth CAP signal is generated by subtracting the I path signal from the Q path signal, which is given by:

(4) $$s_{n}\left(t\right)=D_{In}*g_{In}\left(t\right)-D_{Qn}*g_{Qn}\left(t\right)$$

where ∗ is the time-domain convolution. The MultiCAP signal is generated by summing up (4), which is expressed by:

(5) $$s\left(t\right)=\sum_{n=1}^{N}s_{n}\left(t\right).$$

In our demonstration system, the MultiCAP signal is generated in MATLAB and then uploaded to the AWG, which produces a corresponding waveform at a sampling frequency of $f_{T}$ to modulate the LED panel’s intensity.

At the receiver, demodulation is carried out in the reverse order of the modulation. After the flicker removal and resampling operations, the signal y(t), which is captured by the OSC at a sample frequency of $f_{R}$, turns to the signal $s^{\prime }\left(t\right)$, which is ready for demodulation. The signal $s^{\prime }\left(t\right)$ is first applied to *N* pairs of match filters with the pulse impulse response of $g_{In}\left(-t\right)$ and $g_{Qn}\left(-t\right)$, n=1,⋯,N, and then down-sampled to recover the transmitted I/Q symbols. Following QAM constellation demapping, *N* streams of symbols $\left\{D_{n}\prime \right\}$ are obtained and compared with the transmitted data $\left\{D_{n}\right\}$ to calculate the symbol error rate (SER) or the bit error rate (BER). The selection of the QAM order $k_{n}$ is realised using a simple bit-loading algorithm, which iteratively searches for the largest $k_{n}$ that is capable of supporting a BER below the 7% forward error correction (FEC) limit of $3.8\times 10^{-3}$. In our work, the determination of the bit-loading pattern was only carried out at the longest distance (2 m), which has the worst SNR profile and it can then support shorter distance with better BER performance.

Note that the flicker removal is to mitigate the baseline wander that is caused by the current ripple of the LED driver and it will be detailed in the next section. Additionally, since the nominal frequency of $f_{R}$ is not identical to $f_{T}$, a resampling process is applied to match the underlying sampling rate with that of the reference transmitted data. Using the demodulation algorithms that are described above, the recovered data stream is compared with the transmitted data to determine the BER. Table 2 lists the key parameters adopted in this work.

## 3. Challenges and Results

### 3.1. Flicker Issue and the Digital Signal Processing (DSP)-Based Solution

Flicker is a common problem in LED lighting, which is mainly caused by the low-frequency (ranging from 3 Hz to ∼1 kHz) ripple of the current generated by the power driver in most cases [42]. Unlike the precise voltage sources that were used in our measurements, the commercial LED driver converts AC mains to a “noisy” DC current to drive the LED panel. For instance, the full-wave bridge rectifier or power factor correction (PFC) circuitry, which are two typical circuits that have been widely adopted in commercial LED drivers, will generate ripples at twice the AC line frequency [42]. If not suppressed properly, the ripples can lead to severe flicker problems that cascade to data perturbations. Unfortunately, although the driving current from the commercial LED driver has been filtered to prevent the ripples from introducing noticeable flicker, the residual ripple amplitude is still quite high from the communication point of view. There are two major disadvantages: (*i*) the reduced modulation depth for VLC signals—since the VLC signal is superimposed onto the bias current, the headroom left for VLC signals is reduced if the bias current has a high ripple amplitude; (*ii*) the low-frequency flicker can interfere with the signal falling in the frequency range. The following two solutions help to combat the flicker issue:

1. A high-pass filter circuit can be inserted to filter out the low-frequency flicker if it is possible to modify the analogue frontend of the optical receiver.
2. If it is not feasible to change the hardware, then DSP techniques can be used to remove the flicker by subtracting the low-frequency “envelope” from the received signal.

**Figure 8:**
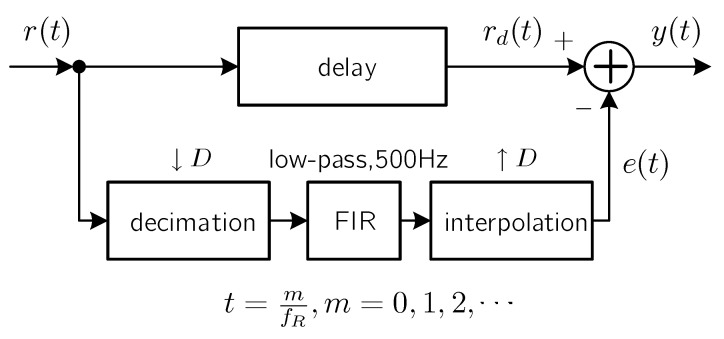
Digital signal processing (DSP)-based solution to remove the flicker in the received signal. r(t) is the signal captured directly by the oscilloscope (OSC) at a sample rate of $f_{R}$ and e(t) is the estimated low-frequency “envelope” signal for the flicker. y(t) is flicker-free after the removal of e(t) from r(t).

The first approach requires a hardware modification according to the specification of flicker and it is not always feasible, while the second DSP approach is more flexible and it can be applied universally. Figure 8 illustrates the detailed block diagram of the DSP solution. The fundamental idea is to subtract the estimated flicker signal from the received signal r(t). A low-pass finite impulse response (FIR) filter is used to filter out the low-frequency components in order to recover the flicker signal. Before feeding r(t) through the FIR filter, a decimation operation, or downsampling, is applied to decrease the sampling rate to reduce the implementation complexity of the succeeded FIR filter. After the FIR filter, an interpolation operation is paired to increase the sampling rate back to the original sampling rate.

**Figure 9:**
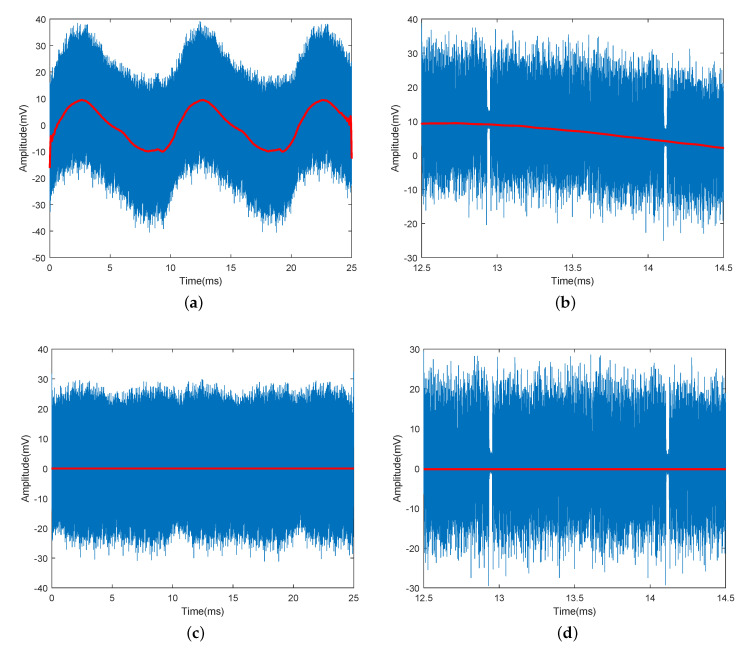
(**a**) Received signal r(t) (blue) and the corresponding estimated flicker signal e(t) (red); (**b**) a zoom-in view of a segment of r(t) and e(t); (**c**) filtered signal y(t); (**d**) a zoom-in view of a segment of y(t).

**Figure 10:**
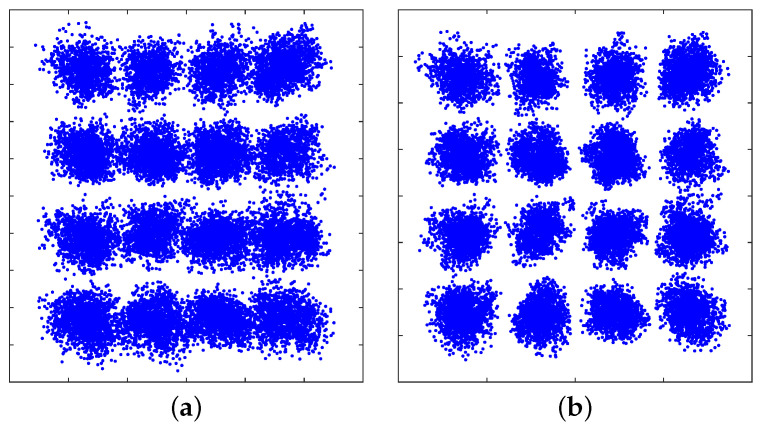
(**a**) Decoded constellation diagram at the 1st carrier before flicker removal; (**b**) Decoded constellation diagram at the 1st carrier after flicker removal.

Figure 9a shows the signal r(t) in blue, which is captured by the OSC and exhibits a slowly changing envelope that is caused by the flicker. From the waveform, the period of the flicker can be roughly estimated to be around 10 ms, indicating a fundamental frequency of 100 Hz (twice the UK mains frequency). Because the flicker is not a perfect single-tone sine wave, a low-pass FIR filter with a cut-off frequency of 500 Hz is used to let a frequency up to the 5th harmonic pass through to recovery a close replica of the flicker. The filtered signal e(t) is shown in red in Figure 9a. Figure 9b also provides a zoom-in view of r(t) and e(t). It can be observed that the flicker e(t) has been successfully extracted from r(t) and it corresponds to the fluctuating DC level. Once subtracted from r(t), the flicker can be removed, and a stable DC level at around zero is achieved, as shown in Figure 9c,d. The improvement in BER performance can be clearly observed from the decoded constellation diagrams at the 1st carrier before and after flicker removal, as shown in Figure 10. We also find that only the 1st carrier was affected, which confirms our analysis that the flicker interferes with the sub-band while using the same spectrum for data transmission.

### 3.2. Results and Discussion

**Figure 11:**
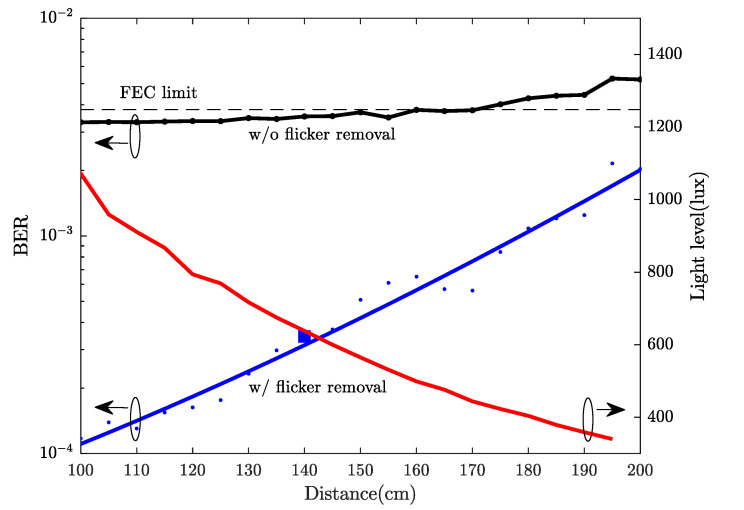
Bit error rate (BER) performance and light level vs. distance.

The BER was measured at varying distances from 1 to 2 m, see Figure 11. For reference purposes, a light level (in lux) curve is also provided. The experimental results show that, with the proposed flicker removal DSP solution, a 40 Mb/s link can be supported up to 2 m with an illuminance level of ∼300 lux (minimum illuminance for indoor workplaces in accordance with EN 12464-1 [43]), maintaining a BER below the 7% FEC limit. When the flicker removal algorithm was not applied, the system performance was mainly limited by the BER performance of the 1st sub-band and the overall BER level was close to the FEC limit with a narrow margin. The effectiveness of the flicker mitigation was validated by the improvement in the system performance and robustness.

**Figure 12:**
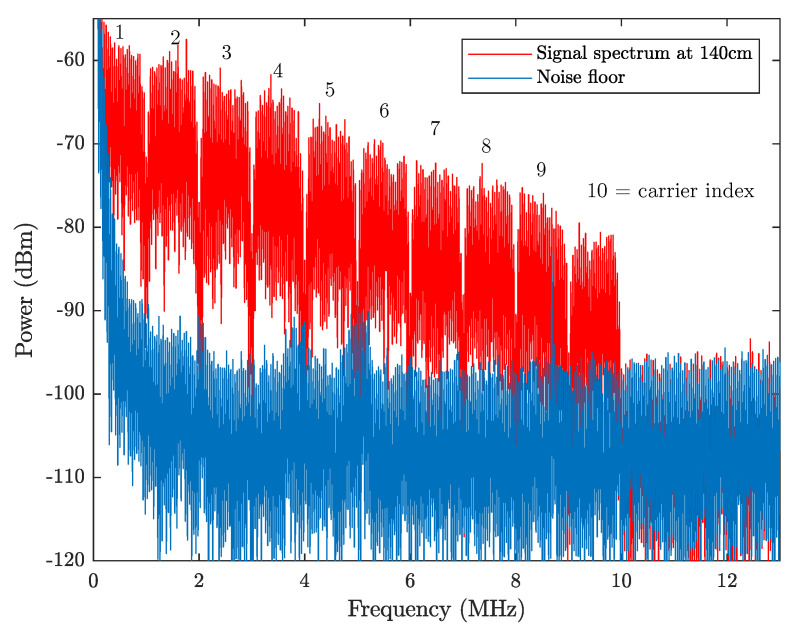
Measured signal spectrum and background noise spectrum at the distance of 1.4 m.

**Figure 13:**
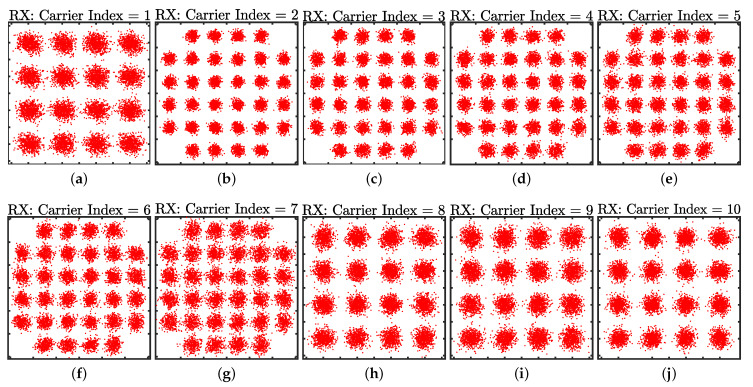
Demodulated constellation diagrams at the distance of 1.4 m for the (**a**) 1st carrier; (**b**) 2nd carrier; (**c**) 3rd carrier; (**d**) 4th carrier; (**e**) 5th carrier; (**f**) 6th carrier; (**g**) 7th carrier; (**h**) 8th carrier; (**i**) 9th carrier; (**j**) 10th carrier.

**Figure 14:**
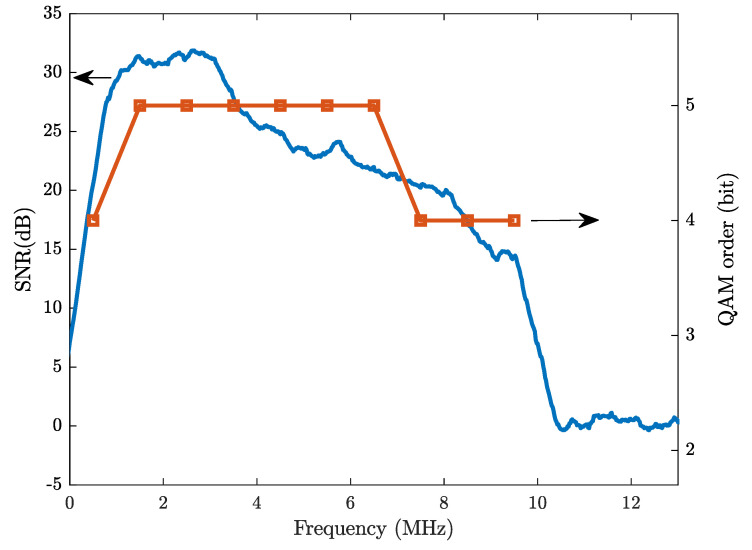
The measured signal-to-noise ratio (SNR) profile and the bit-loading pattern at the distance of 1.4 m.

In order to better demonstrate the system performance, the measured spectra of the received signal and background noise at a distance of 1.4 m (with a light level of 600 lux measured) are shown in Figure 12. The ten carriers can be clearly observed, with every recovered constellation diagram from each carrier included in Figure 13. The corresponding SNR curve is given in Figure 14 with the bit-loading pattern being plotted. As mentioned earlier, the bit-loading pattern is determined at the longest distance and, therefore, the SNR profile at the distance of 1.4 m can support quite clear constellation diagrams, as shown in Figure 13. A video demonstrating the successful demodulation of the 10th carrier is also provided as Supplementary Materials for interested readers.

Two major limiting factors for achieving higher $R_{b}$ using the off-the-shelf LED panel are observed. Firstly, the modulation bandwidth is limited to 10 MHz, because severe distortion, which might be attributed to the LED’s nonlinearity and the parasitic in the electronics, is incurred beyond 10 MHz. It could be a big issue when trying to reuse the existing lighting sources in the applications of high-speed VLC standards, like G.9991 [18] requiring a minimum bandwidth of 50 MHz. Secondly, the flicker that is caused by the LED driver inevitably reduces the modulation depth or the transmitted optical power for VLC signals, imposing a limit on the SNR and the achievable $R_{b}$. An improvement in data rates is expected if the LED driver is optimised for VLC.

## 4. Conclusions

The feasibility of upgrading the existing indoor LED-based lighting infrastructure with add-on VLC functionality was validated by our demonstration system. With a bias-tee, a minimum modification was applied to the large LED panel that was installed in the ceiling to enable intensity modulation for simultaneous communication and illumination. A transmission rate of 40 Mb/s was supported up to 2 m with a measured illuminance level of ∼300 lux. The key factors preventing our system from achieving higher data rates are the limited bandwidth of the LED panel and the flicker that is caused by the low-cost LED driver. To combat those issues, we utilised the low-complexity MultiCAP modulation to exploit the spectrum out of the 3-dB bandwidth and proposed a DSP-based solution to remove the baseline wander effect that is caused by the flicker.

## Figures and Tables

**Table 1:** The list of equipment.

Item	Value
LED panel model	DFx 563-004-01, 4000 K, 36 W
Lumens	2700 lm
Luminous Efficiency	75 lm/W
Voltage source	Keysight E3631A
Arbitrary waveform generator	Tektronix AFG3022
Spectrum analyser	Keysight N9020A

**Table 2:** Key system parameters.

Item	Value
Oscilloscope	Keysight DSO9254A
Arbitrary waveform generator	Tektronix AFG3022
Arbitrary waveform generator output voltage	10 Vpp (set at the 50 Ω impedance)
Modulation bandwidth	$B_{\mathrm{mod}}$ = 10 MHz
MultiCAP carrier number	*N* = 10
Carrier frequencies	0.5 to 9.5 MHz with a step size of 1 MHz
Square-root raised cosine filter roll-off factor	β=0.15
Square-root raised cosine filter length	20 symbols
Baseband symbol rate	$R_{s}=1/T_{s}=$ 869.57 kBaud
Bit-loading pattern	$\{k_{n},n=1,\cdots ,N\}=$ {4 5 5 5 5 5 5 4 4 4}
Aggregate data rate	$R_{b}=\sum_{n=1}^{N}R_{s}k_{n}=40$ Mb/s
Distance	Up to 2 m

## Data Availability

The data presented in this study are available on request from the corresponding author.

## Supplementary Materials

The following are available at https://www.mdpi.com/1424-8220/21/5/1697/s1, Video S1: vlc-panel-demo-multicap-last-carrier.
